# Baseline Ultrasound Assessment Improves the Response to Apremilast in Patients with Psoriatic Arthritis: Results from a Multicentre Study

**DOI:** 10.31138/mjr.271223.bua

**Published:** 2024-12-31

**Authors:** Antonella Farina, Patrizia Del Medico, Simone Parisi, Andrea Becciolini, Elisa Visalli, Aldo Biagio Molica-Colella, Federica Lumetti, Rosalba Caccavale, Palma Scolieri, Romina Andracco, Francesco Girelli, Elena Bravi, Matteo Colina, Alessandro Volpe, Aurora Ianniello, Veronica Franchina, Ilaria Platè, Eleonora Di Donato, Giorgio Amato, Carlo Salvarani, Gianluca Lucchini, Francesco De Lucia, Ylenia Dal Bosco, Francesco Molica Colella, Daniele Santilli, Giulio Ferrero, Antonio Marchetta, Eugenio Arrigoni, Michele Riva, Rosario Foti, Gilda Sandri, Vincenzo Bruzzese, Marino Paroli, Enrico Fusaro, Alarico Ariani

**Affiliations:** 1 Rheumatology Outpatient Clinic, Internal Medicine Unit, Ospedale “A. Murri”, Fermo, Italy;; 2 Rheumatology Outpatient Clinic, Internal Medicine Unit, Civitanova Marche Hospital, Civitanova Marche, Italy;; 3 Rheumatology Department, Azienda Ospedaliera Universitaria Città della Salute e della Scienza di Torino, Torino, Italy;; 4 Internal Medicine and Rheumatology Unit, University Hospital of Parma, Parma, Italy;; 5 Rheumatology Unit, Policlinico San Marco University Hospital of Catania, Catania, Italy;; 6 Rheumatology Unit, Azienda Ospedaliera Papardo, Messina, Italy;; 7 Rheumatology Unit, Azienda USL of Modena and University Hospital “Policlinico di Modena”, Modena, Italy;; 8 Department of Biotechnology and Medical-Surgical Sciences, Sapienza University of Rome, Polo Pontino, Latina, Italy;; 9 Rheumatology and Gastroenterology, Department of Internal Medicine, “Nuovo Regina Margherita” Hospital, Rome, Italy;; 10 Internal Medicine Unit, Imperia Hospital, Imperia, Italy;; 11 Rheumatology Unit, Ospedale GB Morgagni - L Pierantoni, Forlì, Italy;; 12 Internal Medicine and Rheumatology Unit, Ospedale G. Da Saliceto, Piacenza, Italy;; 13 Internal Medicine and Oncology Unit, Ospedale Santa Maria della Scaletta, Imola, Italy;; 14 Unit of Rheumatology, IRCCS Sacro Cuore Don Calabria Hospital, Negrar, Verona, Italy;; 15 Rheumatology Outpatient Unit, ASL Novara, Italy;; 16 UOC Oncologia Medica Azienda Ospedaliera Papardo, Messina, Italy;; 17 Rheumatology Unit, University of Modena and Reggio Emilia, Azienda Ospedaliero-Universitaria, Policlinico di Modena, Modena, Italy;; 18 Internal Medicine Unit, University of Milano-Bicocca, Milano, Italy;; 19 Unit of Diagnostic and Interventional Radiology, Santa Corona Hospital, Pietra Ligure, Italy

**Keywords:** apremilast, psoriatic arthritis, personalised medicine, musculoskeletal ultrasonography

## Abstract

**Background:**

Psoriatic arthritis (PsA) phenotypes show different responses to the many available drugs. For a tailored medicine, it is important to choose the most effective treatment according to patients’ characteristics. Apremilast is recommended in PsA with moderate activity. In clinical practice, the most suitable PsA patients for apremilast are those affected by the peripheral oligo-articular arthritis. However, it is not so straightforward to definitely identify this phenotype. Musculoskeletal ultrasound (MUS) is a good tool for detecting the joints actually involved by PsA. The aim of this study is to verify if MUS assessment is useful in selecting the best PsA responders to apremilast.

**Methods:**

The following data of all consecutive PsA patients from 15 centres were recorded: anamnestic data, disease activity, PsA phenotype, apremilast treatment duration and reason of suspension. MUS assessment before apremilast treatment was the criteria which clustered patients in two groups. Apremilast retention rate estimate the drug’s effectiveness. The Cox analysis revealed the risk factors associated with treatment persistence. Mann-Whitney U and Chi-squared tests assessed the intergroup differences.

**Results:**

Only 40% of 356 patients (M:F: 152/204; median age 60 yrs) received MUS examination. In MUS group the moderate disease (median DAPSA 22.9 vs 26.9; p=0.0006) and the oligo-articular phenotype (63.6% vs 36.1%, p<0.0001) were more common. The retention rate was higher in MUS group (HR 0.55 IC95% 0.32–0.94; p=0.03).

**Conclusion:**

In apremilast treated PsA patients, baseline MUS assessment is related to an increased retention rate. MUS may identify patients’ characteristics favourable to apremilast response.

## INTRODUCTION

Psoriatic arthritis (PsA) is a chronic, systemic inflammatory disease affecting 0.3–1.0% of the general population.^1^ Current PsA treatments include conventional synthetic DMARDs (csDMARDs), biologic DMARDs (bDMARDs) and small molecules, such as apremilast, an oral PD4 inhibitor.^2^ The availability of drugs with so many different mechanism of action, push the rheumatologists to think about the best pharmacological strategies based on patients’ characteristics.^3,4^

The GRAPPA guidelines suggest an approach based on disease domains: peripheral arthritis, axial arthritis, enthesitis, dactylitis, and skin and nail disease.^5^ Even according to ACR/EULAR recommendations the main driver of treatment choice should be the predominant clinical PsA manifestation: poly-articular, oligo-articular, enthesitis, or axial disease.^6^ For example, the apremilast effectiveness is mainly found in patients with mild disease (i.e. oligoarticular disease or low disease activity).^7^

The musculoskeletal ultrasound (MUS) is a convenient, non-invasive, and cost-effective imaging technique providing important elements for PsA management.^8^ In particular, it distinguishes the joints affected by synovitis (presence of effusion and power Doppler signal) from those damaged without activity signs (irregularity of the bone profile; synovitis without power Doppler).^9^ It is reasonable to suppose that MUS can better identify joints affected by active disease making the articular count more accurate.^10,11^ In case of an apremilast, MUS evaluation before treatment may lend the candidate a benefit in terms of clinical response.

The main objective of this study is to evaluate whether in a cohort of PsA patients treated with apremilast, baseline MUS has any influence on the drug effectiveness.

## METHODS

### Patients

The analysed population is part of the observational retrospective study BIRRA (BIologics Retention Rate Assessment).^7^ The study is carried out following the Declaration of Helsinki principles and approved by the Comitato Etico dell’Area Vasta Emilia Nord (protocol code 34713, approved on 28 August 2019). All PsA patients (diagnosed according to ClASsification criteria for Psoriatic ARthritis- CASPAR).^12^ treated with apremilast, from fifteen Italian rheumatological referral centres were consecutively included.

Some patients, in accordance with physician decision based on clinical involved joints, underwent MUS examination.

Patients who received a MUS evaluation just before starting the apremilast treatment, formed the MUS assessed group (MAG); the others pooled the clinical assessed group (CAG).

### Data

For each patient, the following data were recorded: general characteristics (age, sex, body mass index [BMI], smoking habit, and date of PsA, psoriasis onset, and diagnosis), PsA disease activity (number of tender/swollen joints, painful enthesis and fingers affected by dactylitis, C-reactive protein, pain Visual Analog Scale [VAS], and Patient Global Assessment [PGA], values), apremilast related information (date of the first and last treatment administration), possible reason for discontinuation, presence of relevant comorbidities (TB infection, HCV, HBV, or malignancy), and previous and concomitant treatments. According to the number of clinically affected joints established if there was an oligo-articular or poly-articular phenotype.

### Musculoskeletal Ultrasound Assessment

**Figure 1.**
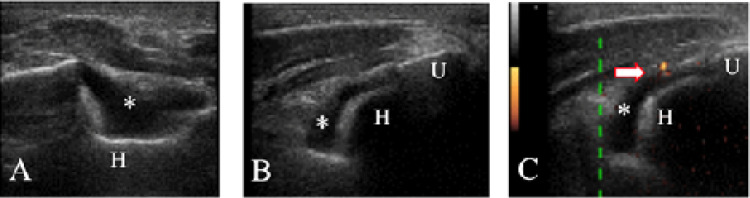
Examples of joints with inflammatory features. **(A)** Psoriatic arthritis. Transversal scan of the posterior joint recess of the elbow. Synovitis of the elbow (*). **(B)** Psoriatic arthritis. Longitudinal scan of the posterior joint recess of the elbow. Synovitis of the elbow (*). **(C)** Psoriatic arthritis. Longitudinal scan of the posterior joint recess of the elbow. Presence of power doppler signal in the elbow (arrow). H: humerus; U: ulna (olecranon process).

Ultrasound assessment was performed by rheumatologists with at least 10-years’ experience in musculoskeletal ultrasound. The systematic multi-planar greyscale and power Doppler examination of joints (both clinically involved or not) was performed by using multi-frequency linear array transducer (6–18 MHz).^13^ Synovial effusion, synovial hypertrophy, and power Doppler, as defined by OMERACT,^14^ identified the joints with inflammatory features (**Figure 1**).

### Statistical Analysis

Mann-Whitney U test (for continuous variables) and Chi-squared test (for categorical variables) detected the differences between the two groups. The retention rate is the best estimator of drug’s clinical effectiveness.^15^ Adjusted Survival curve graphically shows the apremilast retention rate in both groups,^16^ while the Cox analysis revealed which of the following were the factors associated with treatment persistence: age, sex, BMI, smoke habit, disease duration, relevant comorbidities (i.e. TB infection, HCV, HBV, or malignancy), disease activity scores (DAPSA, LEI and Dactylitis- number of fingers), csDMARDs association, previous bDMARDs and MUS evaluation before apremilast treatment. Statistical significance was achieved if p-value was < 0.05. All analyses were performed using Jamovi statistical software, version 2.3.21.0.

## RESULTS

The baseline characteristics of the whole cohort were already reported.^7^ About 40% (140/356) of patients underwent MUS at baseline. The differences between CAG and MAG are shown in **Table 1**. In general, in MAG there was a lower disease activity (taking into account only the peripheral arthritis, i.e. DAPSA) with a higher prevalence of dactylitis and enthesitis. CAG patients were a bit older and with a history of less use of pharmacological treatments (both previous bDMARDs and concomitant csDMARDs). The two groups were different even from the point of view of the prevalence of oligo-articular phenotype. The MAG and CAG groups showed different median persistence on apremilast treatment, respectively 24.1 (IQR 11.1–36.6) months vs 13.6 (IQR 5.6–30.10) months.

**Table 1.** Clinical assessment group (CAG) and MUS assessment group (MAG) baseline characteristics.

	**CAG**	**MAG**	**p-value**
**N**	216	140	-
**Age, median [IQR] (yrs)**	61 [54–69]	58 [50–65]	0.0016
**Sex, (M: F)**	85:131	67:73	nss
**Smokers (%)**	13.8	17.9	nss
**BMI, median [IQR], (kg/m^2)**	25.7 [23.4–29.8]	26.1 [23.7–29.0]	nss
**PsA duration [IQR], median (months)**	44 [13–95]	37 [12–78]	nss
**PsO duration [IQR], median (months)**	13 [0–83]	30 [0–93]	nss
**Relevant comorbidities (%)**	47.7	39.3	nss
**Tender joints, median [IQR]**	8 [4–12]	4 [3–7]	<0.000001
**Swollen joints, median [IQR]**	3 [2–5]	2.5 [2–4]	0.0434
**CRP [IQR], median (mg/dl)**	2.9 [0.8–5.2]	1.0 [0.7–3.0]	0.0057
**DAPSA [IQR], median**	27.0 [20.4–34.2]	22.9 [18.2–29.0]	0.0004
**LEI** ≥**1(%)**	35.6	65.0	<0.001
**Dactylitis – fingers** ≥**1 (%)**	29.2	42.1	0.012
**csDMARDs association (%)**	13.4	27.1	0,0012
**Previous bDMARDs (%)**	19.4	31.4	0,0100
**Oligo-articular phenotype (%)**	36.1	63.6	<0.0001

CAG: clinical assessment group; MAF: MUS assessment group; nss: not statistically significant; IQR: interquartile range; BMI: body mass index; PsO: psoriasis; PsA: psoriatic arthritis; CRP: C-reactive protein; DAPSA: Disease Activity index for PSoriatic Arthritis; LEI: Leed Enthesitic Index.

**Figure 2.**
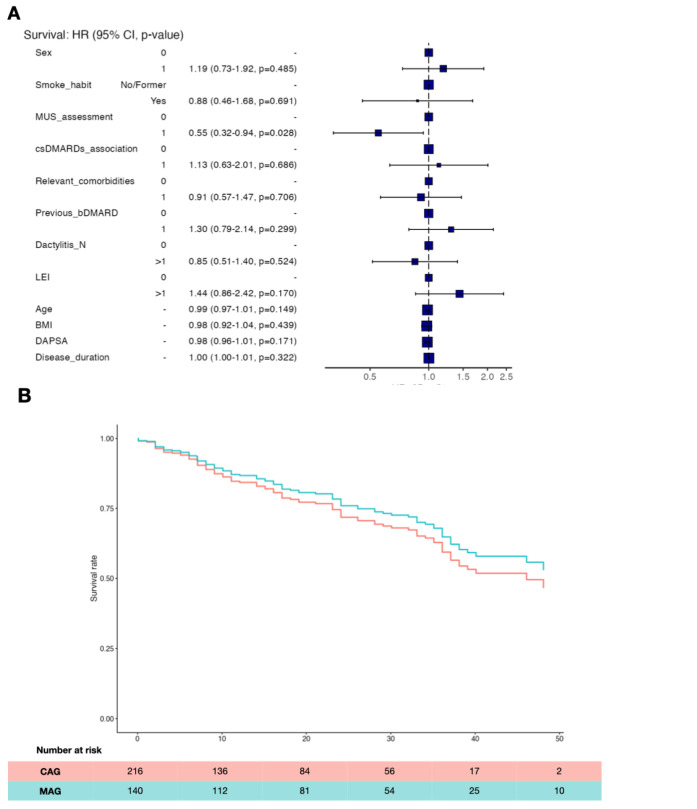
Hazards Regression Plot **(A)** and Kaplan-Meier curve adjusted for MUS basal assessment **(B)**. CAG: clinical assessment group (red); MAG: MUS assessment group (green); time in months.

Among the above-mentioned risk factors, the only one influencing the retention rate was the baseline MUS assessment (Hazard ratio 0.55 95% CI 0.32–0.94; p=0.028) (**Figure 2A**).

The Adjusted (for MUS basal assessment) Kaplan-Meier curve shows that the apremilast retention rate difference between MAG and CAG is about 3% after the first year. Moreover, in the following three years the delta increases of 1% per each year (**Figure 2B**).

## DISCUSSION

As far as we know, this is the first study investigating whether MUS can contribute to a tailored medicine strategy in the setting of PsA. The MUS assessment, performed just before apremilast treatment, clustered real-life PsA patients into two groups (i.e. MAG e CAG). Although both groups had a consistent and comparable number of patients (140 vs. 216), they differed significantly in terms of baseline disease activity, previous/concomitant treatment, and prevalence of the oligo-articular phenotype.

Apremilast is recommended in PsA with moderate activity^6^ and there is a wide agreement that in clinical practice should be reserved to patients with oligo-articular involvement.^4,17^ Previous analysis from our cohort demonstrated that this phenotype is associated to long-lasting apremilast treatment and achievement of low disease activity or remission at 12 months.^7,18^

MUS plays a relevant role in portraying PsA patients.^8,11^ It enables rheumatologists to confirm the inflammatory involvement of clinically detected joints^9^ and can even detect subclinical synovitis in PsA patients in remission, who may experience a short-term arthritis flare.^10^ Thus, it is reasonable to wonder whether MUS assessment, by detecting asymptomatic inflammation, can help reclassify the PsA phenotype from oligo-articular to poly-articular.^19^ In other words, MUS support may aid clinicians in better identifying the oligo-articular subset of patients who are more responsive to apremilast.

In our cohort, the higher apremilast retention rate and oligo-articular prevalence in MAG compared to CAG are correlated. Moreover, MAG have a history of disease with a heavier pharmacological burden (both previous and concomitant therapy are more common than in CAG). This aspect may have encouraged the rheumatology to better understand the actual disease manifestations. Enthesitis is far more common in MAG, which can reasonably be attributed to MUS assessment.^20^ All these findings support the hypothesis that MUS is an essential tool in accurately identifying all PsA manifestations (including the peripheral arthritis subset), improving and customising the therapeutic decision process.

The findings of this ancillary observational study do not allow further elaboration on this topic. None of our data rule out the possibility that MUS assessment represents only indirect evidence of the rheumatologist’s best effort to explore PsA manifestations and customise patient’s treatment. We acknowledge other limitations, such as the absence of a control group (i.e. PsA patients starting other DMARDs). Additionally, we did not report specific MUS alterations, such as synovitis, presence of power-Doppler, tendon thickening, or irregularity, making it impossible to verify if a specific MUS pattern was more common and/or associated with treatment withdrawal. Moreover, we have no data regarding MUS assessment in the follow-up period. Furthermore, patients’ reported outcomes about skin and psychological dimensions were completely neglected, preventing us from establishing their impact size (if present). However, despite the lack of high-quality evidence about the role of MUS in identifying the best PsA responders to apremilast, some findings still support this working hypothesis.

In conclusion, baseline MUS appears to be helpful in selecting PSA patients who are responsive to apremilast. Therefore, the hypothesis that baseline MUS can be a useful tool in tailored medicine seems worthy of further investigations.

## Data Availability

The data underlying this article will be shared on reasonable request to the corresponding author.

## PRESENTATION

Preliminary results have previously been presented (Abstract Book) at EULAR 2023.
